# Influence of thermal and mechanical fatigue on the shear bond strength of different all-ceramic systems

**DOI:** 10.4317/jced.53728

**Published:** 2017-08-01

**Authors:** Hugo-Alberto Vidotti, Jefferson-Ricardo Pereira, Elizeu Insaurralde, Luiz F. Plaça, José R. Delben, Accácio-Lins do Valle

**Affiliations:** 1 Department of Prosthodontics, University of São Paulo, Bauru, SP, Brazil; 2 Department of Prosthodontics, University of Southern Santa Catarina, Tubarão, SC, Brazil; 3 Department of Prosthodontics and Restorative Dentistry, School of Dentistry, University of South Mato Grosso, Campo Grande, MS, Brazil; 4 Department of Physics, Center of Exact Sciences and Technology, University of South Mato Grosso, Campo Grande, MS, Brazil; 5 Department of Physics, Center of Exact Sciences and Technology, University of South Mato Grosso, Campo Grande, MS, Brazil; 6 Department of Prosthodontics, University of São Paulo, Bauru, SP, Brazil

## Abstract

**Background:**

To evaluate the influence of thermal and mechanical fatigue on the shear bond strength of different all-ceramic cores and veneering porcelain interfaces.

**Material and Methods:**

All-ceramic systems tested were lithium disilicate and zirconia veneered by layering technique. Sixty specimens (n=20) were subjected to shear bond strength. Ten of them were thermal and mechanical cycled. Fracture analysis was performed with stereomicroscopy and scanning electron microscopy. Energy dispersive X-ray spectroscopy analysis was performed across core/veneer interfaces.

**Results:**

Thermal and mechanical cycling did not influence on bond strength. However, there was significant difference among systems (<0.01). CoCr group presented the highest values, followed by lithium disilicate, and zirconia. Failure modes were predominantly adhesive for CoCr, cohesive in core for lithium disilicate, and cohesive in veneer for zirconia. Energy dispersive X-ray showed interaction zone for CoCr and lithium disilicate groups and was inconclusive for zirconia. Fatigue had no influence on bond strength of groups tested.

**Conclusions:**

The results suggest that there is a chemical bond between core and veneer materials for CoCr and lithium disilicate groups.

** Key words:**Ceramics, electron microscopy, fatigue, mechanical stress, shear bond strength.

## Introduction

Lithium disilicate, a reinforced glass ceramic, and yttria-stabilized tetragonal zirconia polycrystalline (YTZ-P) have been widely used as materials for core of bilayered restorations (1).

The bonding between core and veneer material has an important role to success of all-ceramic restorations. Indeed, resistance to outbreak and growth of a flaw on all ceramic restoration is multifactorial and also depends on the materials composition, thickness and design of the restoration, luting techniques, residual stresses induced by the thermal processing or polishing and differences of elastic module of the restoration components (2).

Clinically, one of the technical complications reported in the YTZ-P reconstructions is chipping or delamination of the veneering porcelain (3,4) and fractographic analysis from clinical failure shows that the crack origin of some fractured restorations was located at the core veneer interface (5). The estimated stress at failure for delamination (26.8 MPa) is higher than the calculated zirconia veneer bond strength reported in previous studies using bond strength test (15-24 MPa) (6,7), and suggests that the core-veneer interface is a critical factor for the success of layered restorations.

Studies have focused on the bond strength of different ceramic core/veneer combinations and suggest that lithium disilicate have higher bond strength to veneering porcelain compared to zirconia (8-11). However, the performance of this interface when subjected to thermal and mechanical induced stresses, like chewing and ingesting hot food or cold drink, is not yet well understood (12-14).

Spectroscopy analyses are useful tools for the interface characterization of inorganic materials. Morphological and elemental composition of the metal–ceramic interfaces by Scanning Electron Microscopy (SEM) and X-ray Energy Dispersive Spectroscopy (EDS) analysis operating in line scan mode is well described in literature (15,16) and had an important rule on the understanding of bonding mechanisms and atomic diffusion between these materials during sintering process. On the other hand, little information is available regarding all-ceramic materials interfaces. SEM, Confocal Raman Spectroscopy and EDS data strongly suggests that there is an interdiffusion zone at YTZ-P/veneer ceramic interface (17-19). There is not, to the authors’ knowledge, such data regarding lithium disilicate core/veneer interface on the literature. Since the influence of thermal and mechanical cycling in conjunction have also not yet been reported, the objective of this study was to investigate the influence of thermal and mechanical cycling on the shear bond strength of YTZ-P and lithium disilicate ceramic and their veneering ceramic and to compare the results to a metal-ceramic system.

## Material and Methods

Following the appropriate University Human Research Ethics Board approval, all-ceramic substructure materials tested were pressed lithium disilicate (LD) (IPS e.max Press, Ivoclar Vivadent, Schaan, Liechtenstein) and yttrium-stabilized zirconium oxide (Y-TZP) machined by the CAD/CAM technique (IPS e.max ZirCAD, Ivoclar Vivadent, Schaan, Liechtenstein) (n=20). A glass–ceramic veneer (IPS e.max Ceram) was used for layering of the all-ceramic core materials. An additional metal ceramic alloy (CoCr) (Fit Cast CoCr, Talladium, Valencia, EUA) was used as control and layered with a compatible glass ceramic veneer (IPS Inline, Ivoclar-Vivadent, Schaan, Liechtenstein) (n=20).  Table 1 shows the materials used and their chemical composition. The shear bond strength samples and the results of the Y-TZP and the metal-ceramic groups were part of a previous study performed by our group (20).

**Table 1:**
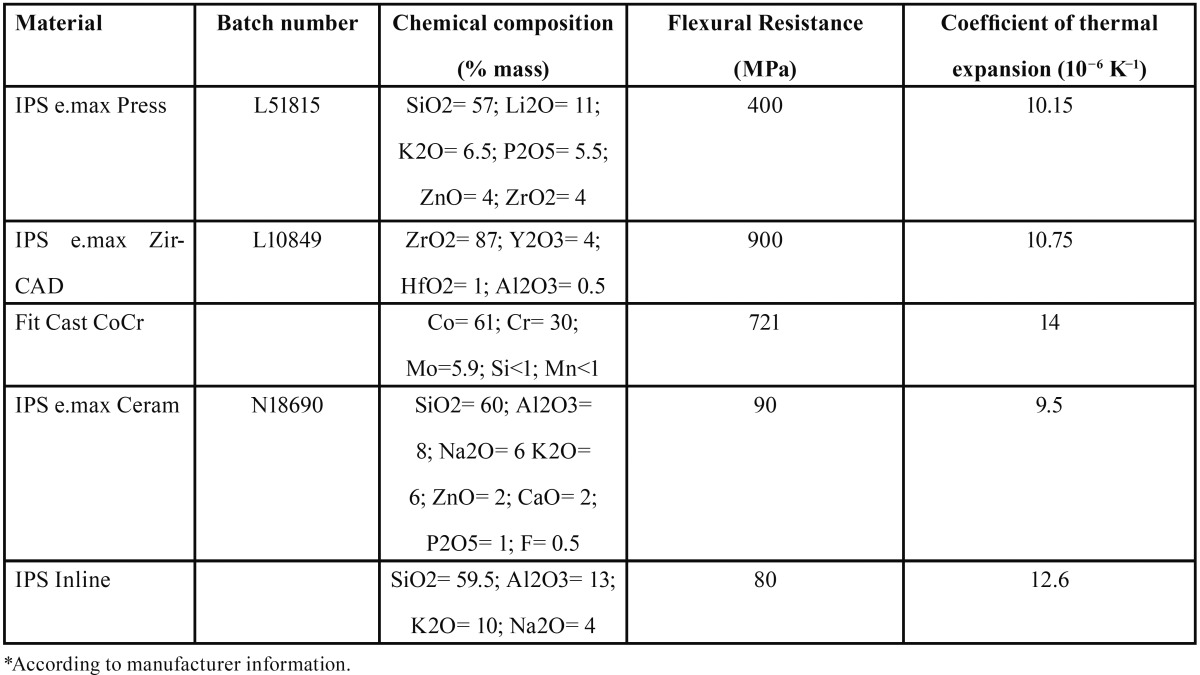
Different materials used in this study, chemical composition and physical properties*.

A cylindrical stainless steel matrix was used for specimen preparation. The same matrix was used for ceramic layering and shear strength testing. The matrix had a central hole with 6.5 mm in depth and 6.0 mm in diameter. A disc (6.0 mm in diameter; 2 mm thick) was used as a spacer to standardize the veneer layer thickness.

The wax patterns were made with the disc positioned inside the perforation. The wax was liquefied at 75˚C and flowed with the aid of a dropper into the perforation. After wax cooling, the patterns were removed by the introduction of the metallic pin in an auxiliary perforation and stored in water until the investment and casting or pressing procedures. For the ZirCAD groups a wax pattern was scanned and patterns were milled in a CAD/CAM system (Cerec InLab, Sirona, Bensheim, Germany).

Veneering was carried out with layering technique as per manufacturer’s instructions for mass preparation, condensing, baking temperature and time.

Half of the specimens from each group (n=10) were subjected to both thermal and mechanical cycling. The other half (10 from each group) were stored in distilled water for 24h at 37˚C prior to shear bond strength test. The specimens were first thermocycled for 6000 cycles between 5 and 55˚C in deionized water (Elquip, São Carlos, Brazil) with dwell time of 15s and transfer time of 5s. After that, specimens were mechanical cycling in aqueous environment at 37˚C with a mechanical cycling machine (Elquip, São Carlos, Brazil) with 3.2 mm diameter indenter inducing 50 N load for 20,000 times with a frequency of 1 cycle per second. The loading was applied axially on the center of the porcelain portion of the specimen.

The samples were positioned into the matrix with the disc at the bottom of the perforation, leaving the ceramic layer visible outside the matrix, such that the shear forces could only be applied at the interface. Shear strength testing was performed in a universal testing machine with a 0.5 mm thick bevel-shaped rod at a crosshead speed of 0.5 mm/min until failure. Data were analyzed statistically by two-way analysis of variance (Anova). Multiple comparisons were made by Tukey’s test. *P*<0.05 was considered statistically significant.

Fracture analysis was performed on stereomicroscope (Stemi 2000-C, Carl Zeiss, Gottingen, Germany) and scanning electron microscope (JSM-6380-LV, JEOL, Tokyo, Japan).

Failure modes were classified as: (CV) cohesive failure in the veneer; (M) mixed fracture starting just near the interface, crossing it and continuing in the core; (A) adhesive failure at the core/veneer interface; (CC) cohesive failure in the core.

One additional specimen from each group was longitudinally sectioned for linear scan EDS analysis. It was embedded in an epoxy resin (Araldite, Basel, Switzerland). After 24h storage in room temperature the specimens were ground with silicon carbide papers (220-2000 grit size) under continuous water cooling. The specimens were ultrasonically cleaned for 10 min in a water bath and sputter-coated with carbon in a sputter-coating unit (SCD 004 Sputter-Coater with OCD 30 attachment, BalTec, Vaduz, Liechtenstein). The interfaces were examined in a scanning electron microscopy (SEM) (JEOL 6830LV Microscope). The elemental distribution across the interfaces was determined by using line scan EDS analysis, with a 18 μm distance crossing the interface.

Statistical analysis was performed using SPSS statistical package (version 18.0, SPSS Inc., Chicago, USA).

## Results

**Figure 1:**
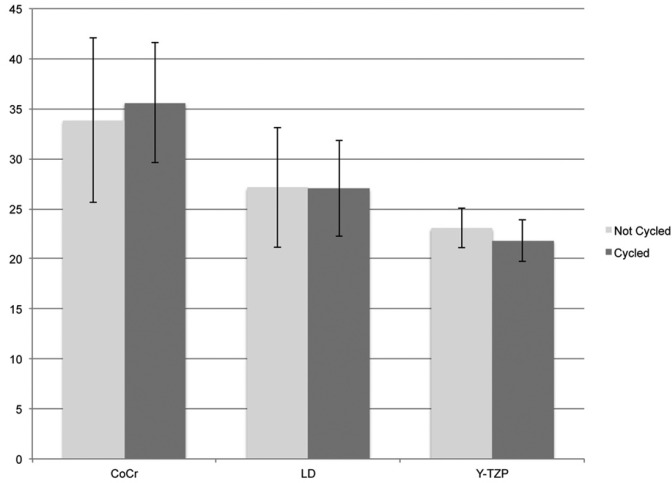
Shear bond strength results with and without aging for the materials combinations tested (MPa). Vertical bars represent the standard deviation.

Results showed similar shear bond strength values before and after thermo and mechanical cycling for all groups (Fig. 1). None of the specimens tested showed debonding during the procedures of aging. The 2-way Anova showed a significant difference for the bond strength among the materials tested at *p*<0.05 ( Table 2). Then, Tukey multiple comparisons test was applied to all the specimens within the same material tested (n=20). The comparisons and the mean shear bond strength for each group are showed in  Table 3. Significant differences between all materials were found.

**Table 2:**
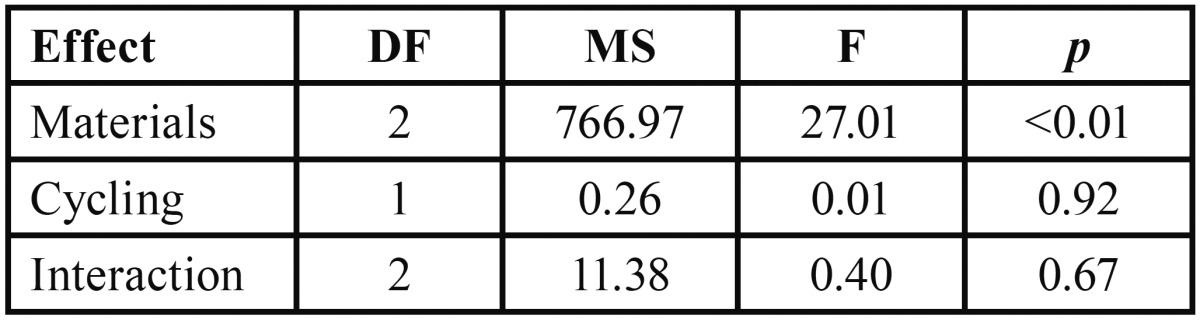
Results of two-way analysis of variance for the cycling fatigue conditions, materials, and interaction of shear bond strength data (*p*<0.05).

**Table 3:**
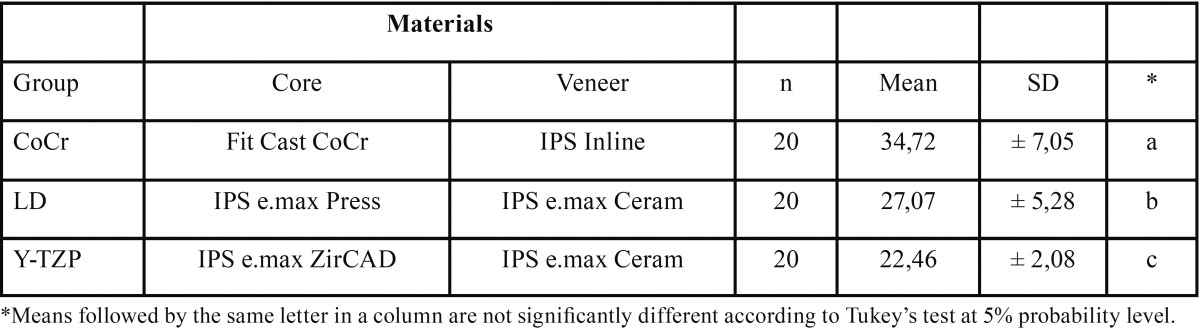
Shear bond strength (MPa) means and standard deviation (SD) and comparisons* between the materials combinations tested.

Fracture analysis results are presented in  Table 4 and distinct failure modes were found for each group. Metal-ceramic group (control) exhibited predominately adhesive failures while lithium disilicate group showed predominately cohesive failure within the core. YTZ-P specimens showed exclusive cohesive failures within the veneering porcelain.

**Table 4:**
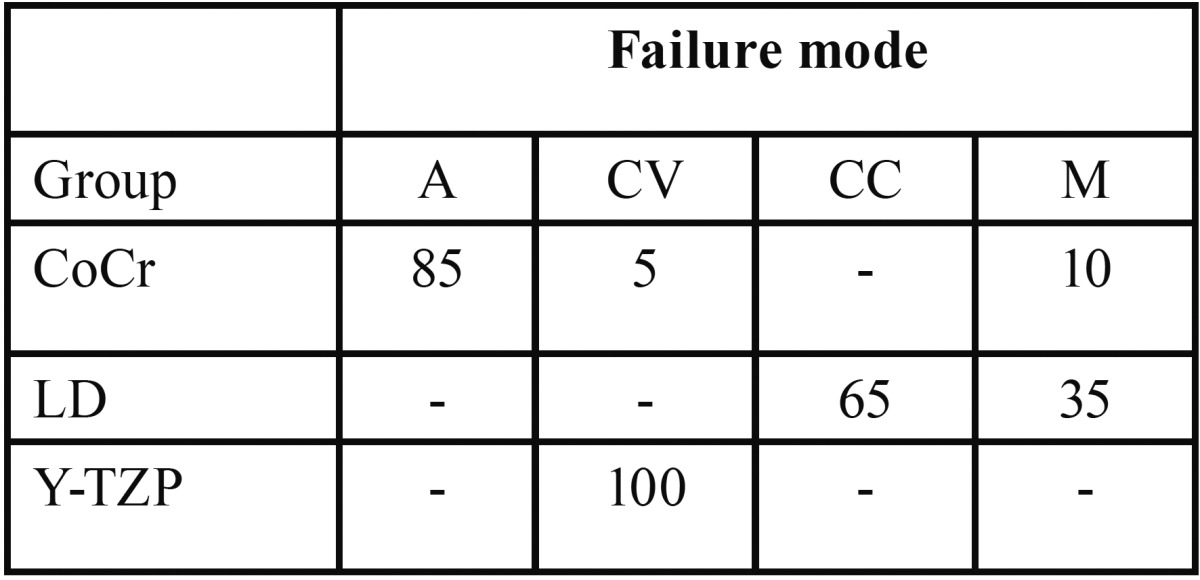
Failure modes for each group (values in %): (CV) Cohesive failure in the veneer, (M) Mixed fracture, (A) Adhesive failure at the core/veneer interface, (CC) Cohesive failure in the core.

**Figure 2:**
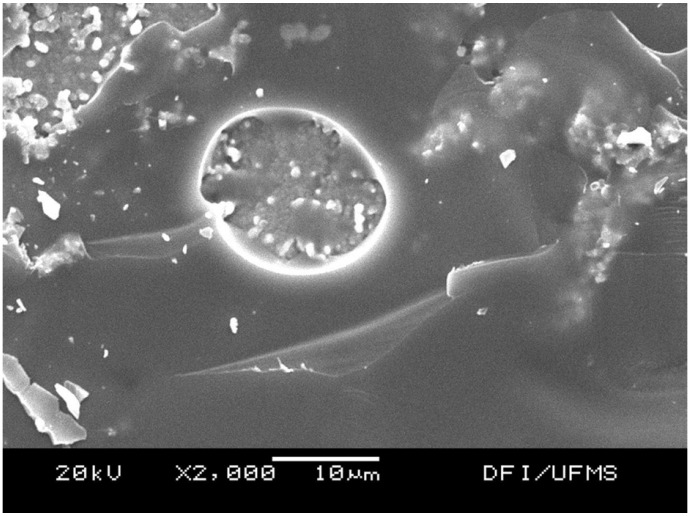
SEM image of Y-TZP group. Cohesive fractures within veneer ceramic were evidenced due to the presence of a thin layer of porcelain attached to core.

Figure 2 shows the Backscattered Electron Images and the linear scan EDS analysis of the different core/veneer interfaces tested. For group CoCr, decreasing curves of Co and Cr densities at metallic phase and increasing Si, K and Al of the glass phase were almost monotonic and sigmoidal. There was a superposition for approximately 4 μm and the curves of Si and Co shows a maximum at the center of the interface while Cr was minimum. For LD group the density variation of elements was smoother and the interdiffusion at interface was approximately 10μm. The core/veneer interface in YTZ-P group is very defined and regular, which indicates a lesser superposition and greater chemical and structural homogeneity of each material. The transition curves of EDS analysis embrace about 3μm.

## Discussion

Regardless of the materials tested, the thermal and mechanical cycling protocol adopted in the present study did not have influence the core/veneer bond strength, thus the first null hypothesis tested was not rejected ( Table 2). Thermo cycling has been used to simulate that condition in in vitro tests and, in some studies, leads to spontaneous debonding of the specimens or reduction of bond strength (12). Mechanical fatigue from of chewing also may contribute to clinical failure, since it subjects restorative materials themselves or their interfaces to repeated stresses. Since no influence of the aging protocol used in the present study was found, it can be concluded that the different interfaces tested resisted the mechanical and thermal stresses to which they were subjected and are able to resist fatigue-challenging situations.

Significant differences between shear bond strength of the materials tested were found. Thus, the second null hypothesis was rejected. The control group (CoCr) showed mean shear bond strength of 34.72 MPa, satisfactory value for metal ceramic interface bond strength according to the International Organization of Standardization that proposed a minimum value of 25 MPa for these systems. Failure mode was predominately adhesive. EDS analysis showed an interaction zone along the interface between the CoCr substrate and the opaque porcelain. It can be inferred that ions inter-diffusion occurs at the interface characterizing a chemically adhesive process predominantly by Si and Co inter-diffusion. Anusavice *et al.* (15) have also described this chemical interaction and suggested that there is an interaction between Cr ions of the Cr2O3 at the core material and the Al provided by the opaque porcelain, forming a mixed complex of Al-Cr-O, which leads to a strong adhesion between the materials.

**Figure 3:**
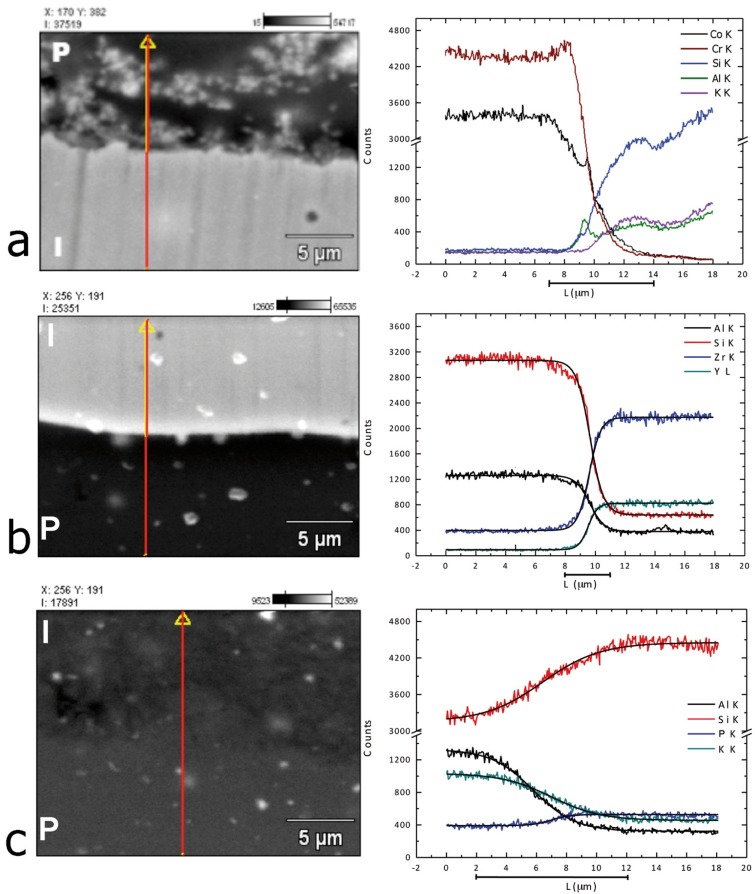
Line scan EDS analysis demonstrating the variation of each element across the different interfaces (a – CoCr; b – YTZ-P; c – LD). The horizontal bars bellow the “X” axis represents the interaction zone.

YTZ-P group showed mean shear bond strength of 22,46 MPa and failure mode predominantly cohesive within the veneering porcelain. Fracture analysis with SEM was mandatory to determine the failure mode, since a very thin layer of veneering porce-lain was attached to the YTZ-P core (Fig. 2) and it was challenging to distinguish it in the optical microscope. SEM images of the interfaces (Fig. 3) showed a well-defined boundary between the two materials. Although EDS analysis showed a possible 3 μm zone of elemental curves variation, it cannot be stated, based on that data, that there is an interaction zone between materials. Since the electron beam has approximately 1μm, it can be considered that the behavior of increasing and decreasing element densities curves may be related to the superposition of the electron beam. Thus, each measure may be a spatial mean of the composition and not necessarily of a real density variation of the elements. Thus, it is not clear, based on the results, if there is an actual chemical interaction between YTZ-P and veneering porcelain. However, other reports (17-19) strongly suggest it. Tholley *et al.* (19) found morphological changes and tetragonal to monoclinic transformation in the zirconia core material in contact with the veneering porcelain that may indicate that there is some sort of chemical interaction between these materials. Durand *et al.* (17) reported, through Raman Imaging Analysis, an interaction zone that ranges from approximately 1.3 μm to 3 μm. The actual chemical interaction mechanisms, however, is yet to be confirmed.

LD group showed mean shear bond strength of 27.07 and failure mode predominantly cohesive within the core similar to the findings of Al-Dohan *et al.* (9) and Ereifej *et al.* (11). SEM images (Fig. 3) showed no defined interface between the materials compared to the other groups and EDS analysis showed an interaction zone of approximately 10 μm. This may be explained by the similar chemical composition and elemental concentration of the core material and the veneering porcelain ( Table 1) that would promote a complete chemical interaction forming a continuum between the two materials, instead of a well-defined interface.

In the present study, thermal and mechanical cycling did not have influence on the shear bond strength of the ceramics systems tested, suggesting that those interfaces has a good stability after induced thermal and mechanical induced stresses.
